# Rollen und Rollendynamiken in der partizipativen Forschungsgemeinschaft

**DOI:** 10.1007/s00103-020-03272-y

**Published:** 2021-01-06

**Authors:** Susanne Kümpers, Sven Brandes, Birte Gebhardt, Christina Kühnemund

**Affiliations:** 1 Fachbereich Pflege und Gesundheit, Hochschule Fulda, Leipziger Str. 123, 36037 Fulda, Deutschland; 2 Landesvereinigung für Gesundheit und Akademie für Sozialmedizin Niedersachsen e. V., Hannover, Deutschland

**Keywords:** Rollen, Partizipative (Gesundheits‑)Forschung, Wissenschaft-Praxis-Kooperation, (Co‑)Forschende, Vertrauen, Roles, Participatory health research, Cooperation between research and practice, Coresearcher, Trust

## Abstract

Aus dem Ansatz der partizipativen Forschung ergeben sich für Forschende und Co-Forschende andere Rollen und Beziehungen als bei traditionellen Forschungskonzepten. Der Artikel beschreibt im Sinne eines Werkstattberichts Reflexionen und Erkenntnisse zu der Frage, wie Rollen im Rahmen partizipativer Gesundheitsforschung (PGF) wahrgenommen und ausgefüllt werden. Wissenschaftler*innen und Praxispartner*innen aus 2 Teilprojekten im Forschungsverbund PartKommPlus werteten dazu ihre Erfahrungen aus mehrjähriger Zusammenarbeit aus und entwickelten Erkenntnisse darüber, welche Dynamiken für die Rollengestaltung wichtig waren und worauf für eine konstruktive Gestaltung der Rollen zu achten ist.

Als 5 wichtige Elemente bei der Entwicklung von Rollen wurden herausgearbeitet: die Bedeutung der Handlungsspielräume, die sich aus den Bindungen an die jeweiligen Institutionen ergeben; die Veränderung der Rollen und Beziehungen über den (Projekt‑)Zeitraum; die Bedeutung von Vertrauen für funktionierende Kooperationsbeziehungen; die Problematik der Vertraulichkeit von Informationen für die wissenschaftliche Verwertung sowie der durch den Veränderungsanspruch partizipativer Forschung begründete politische Charakter der Kooperationen, der geteilte Grundhaltungen und Ziele voraussetzt. Abschließend wird die Bedeutung der gemeinsamen Reflexion der Rollengestaltung herausgearbeitet, die zum Erfolg oder Misserfolg partizipativer Forschungszusammenarbeit sowie zur Qualität ihrer Ergebnisse beiträgt.

## Abstract (en)

The participatory research approach results in changes in roles and relationships for researchers and coresearchers compared to traditional research concepts. In the form of a workshop report, this article describes reflections and findings on the question of how roles are perceived and filled in participatory health research (PHR). Researchers and practitioners from two subprojects in the research consortium PartKommPlus evaluated their experiences from several years of cooperation and developed insights into which dynamics were important for the design of roles and what should be considered for a productive development of roles.

Five important elements were identified: the significance of the scope for action resulting from the ties of the participants to their respective institutions; the change of roles and relationships over the course of the project time; the significance of trust for functioning cooperative relationships; the problem of information confidentiality for scientific exploitation; and the political character of the cooperation, which is based on the claim for change of participatory research and which presupposes shared basic attitudes and goals. Finally, the importance of joint reflection on role design, which contributes to the success or failure of participatory research cooperation and the quality of its results, is highlighted.

## Einleitung und Hintergrund

Die erkenntnistheoretische Position der Partizipativen Gesundheitsforschung (PGF) beinhaltet, nicht über, sondern mit Menschen zu forschen, deren Lebens- oder Arbeitswelt untersucht und ggf. verändert wird [2]. Partizipation der „Co-Forschenden“ – dazu gehören Praxispartner*innen und Community-Partner*innen – soll in allen Phasen des Forschungsprozesses maximiert werden [3]. Idealtypisch wird oft eine annähernde Gleichheit der Beziehungspartner*innen gefordert; an anderer Stelle werden die Forschungsbeziehungen allerdings nicht als gleich („equal“), sondern als gleichberechtigt bzw. angemessen („equitable“) charakterisiert [4, S. 332].

Im wachsenden Fundus der Literatur zu partizipativer Sozial- und Gesundheitsforschung werden auch die spezifischen Rollen^1^ partizipativ forschender Wissenschaftler*innen und der jeweils Co-Forschenden thematisiert. Oft wird die Erweiterung der Rollen der Wissenschaftler*innen reflektiert. Long et al. sprechen von Rollen als „organizer, facilitator, teacher, consultant, supervisor, bringer of money, or provocateur“ [5, S. 252], ohne diese allerdings weiter zu explizieren. In anderen Veröffentlichungen wird lediglich zwischen „professionell Forschenden“ und der „Community“ als Forschungsbeteiligten unterschieden [6]. Weitere Co-Forschende, wie z. B. professionelle Fachkräfte und/oder bürgerschaftlich Engagierte, werden bislang seltener thematisiert. Ähnlich wie in den hier vorgestellten Projekten unterscheiden bspw. Cargo und Mercer [4] zwischen Zielgruppen („end users“), den informellen Unterstützungsnetzwerken der Zielgruppen, der (lokalen) Öffentlichkeit, Fachkräften, die für die Zielgruppe zuständig sind, und im weitesten Sinne politisch Verantwortlichen als von der Forschung direkt oder indirekt Betroffene (S. 331). Die von uns gewählte Unterscheidung zwischen Community-Partner*innen und Praxispartner*innen findet sich auch bei von Unger [7, S. 37]; sie ermöglicht eine pragmatische Unterteilung und weitere Differenzierungen.

Prinzipiell gilt für partizipative Forschungsprozesse, dass Rollen- und Aufgabenverteilung zwischen Wissenschaftler*innen und Co-Forschenden in partizipativen Projekten nicht wie in traditionellen Forschungskontexten klar definiert und voneinander abgegrenzt sind [7, 8]. Wissenschaftler*innen geben ihre Distanz und Neutralität zumindest teilweise auf, indem sie zusammen mit Co-Forschenden an Veränderungen im Feld arbeiten. Praxispartner*innen wiederum begeben sich in die Rolle der Co-Forschenden und somit in das Feld der Wissenschaft.

In diesem Artikel beschreiben wir Erfahrungen mit Rollen und deren Dynamiken im Verlauf von 2 partizipativen Forschungsprojekten im Kontext kommunaler Gesundheitsförderung. Der Artikel beschreibt im Sinne eines Werkstattberichts Reflexionen und Erkenntnisse zu der Frage, wie Rollen im Rahmen partizipativer Gesundheitsforschung (PGF) wahrgenommen und ausgefüllt werden. Während frühere Studien/Arbeiten häufig den Blick auf die Rolle „primärer Zielgruppen“ (Community-Partner*innen) in partizipativen Forschungsprozessen richteten, liegt der Schwerpunkt dieser Arbeit in der Beschreibung und Reflexion von Rollenaushandlungen und -dynamiken zwischen Wissenschaftler*innen und Praxispartner*innen. Praxispartner*innen waren im Kontext der hier beschriebenen Projekte Verwaltungsexpert*innen (aus den Bereichen Soziales, Jugend- und Altenhilfe), weitere Fachkräfte (z. B. Leitung Stadtteilzentrum für Ältere, Lehrkräfte, Erzieher*innen) sowie bürgerschaftlich Engagierte (z. B. Seniorenrat, Vorstand Stadtteilzentrum für Ältere).

Ausgangspunkt für die Arbeit an diesem Artikel waren Erfahrungen in den Steuerungsgruppen von 2 Projekten. Als Wissenschaftler*innen und Praxispartner*innen erlebten wir im Verlauf unserer Projekte vielfältige Rollen und diesbezügliche Zuschreibungen und Erwartungen, die zunächst überwiegend implizit blieben. Im Sinne von (Selbst‑)Verständigungsprozessen, also intersubjektiven Prozessen, in denen Beteiligte Klarheit über eigene Lebenssituationen entwickeln [9], entstand, zunehmend projektübergreifend, ein offener Dialog- und Auswertungsprozess.

Dieser Artikel untersucht vor diesem Hintergrund die Frage, wie Praxispartner*innen und Wissenschaftler*innen in partizipativen Forschungsprojekten ihre Rollen definieren, aushandeln und ausfüllen können und welche Dynamiken, Herausforderungen und Chancen sich daraus ergeben.

Die hier dargestellten Prozesse und Ergebnisse resultieren aus den Projekten „Gesunde Stadtteile für Ältere – Age4Health“ und „PEPBS – Partizipative Evaluation der Präventionskette Braunschweig“, die zum Forschungsverbund für gesunde Kommunen (PartKommPlus)^2^ gehören.

### Projekt „PEPBS“

Gemeinsam mit der Stadt Braunschweig ermittelte das Teilprojekt „PEPBS“ exemplarisch, welche Faktoren ausschlaggebend sind, um lebensphasenorientierte Unterstützungsstrukturen erfolgreich für die Prävention gesundheitsbezogener Armutsfolgen bei Kindern zu gestalten.

Im Projekt PEPBS wurden Bausteine der Präventionskette Braunschweig partizipativ evaluiert, u. a. ein Präventionsprojekt zur Förderung des Übergangs von der Hauptschule in den Beruf. An der Umsetzung der Evaluation waren, neben den Wissenschaftler*innen, auch Schüler*innen, Lehrkräfte und Sozialarbeiter*innen der Hauptschule sowie kommunale Akteure verschiedener Verwaltungsbereiche der Stadt Braunschweig und der Landesschulbehörde in partizipativen Prozessen beteiligt. Die kommunalen Akteure nutzten die Erkenntnisse einer Photovoice-Studie [10] gemeinsam, um das Programm weiterzuentwickeln.

### Projekt „Age4Health“

Das Projekt „Age4Health“ untersuchte und förderte gemeinsam mit Praxispartner*innen sowie weiteren professionellen Akteuren, bürgerschaftlich Engagierten und älteren Bürger*innen in 2 hessischen Kommunen Gestaltungsmöglichkeiten für die Beteiligung älterer Menschen, auch von solchen in schwierigen Lebenslagen, sowie die Entwicklung inklusiver und gesundheitsförderlicher Quartiere.

In beiden Fallstudien (Stadtteil: Kassel-Bettenhausen; ländlicher Raum: Witzenhausen, Werra-Meißner-Kreis) kooperierten Praxispartner*innen (aus Kommunalverwaltung, Stadtteilzentrum sowie bürgerschaftlich Engagierte) und Wissenschaftler*innen in Steuerungsgruppen, in denen sie gemeinsam Prozesse initiierten, organisierten und auswerteten.

Von regelmäßigen Runden Tischen ausgehend konnten vielfältige Aktivitäten und Initiativen gemeinsam mit Akteur*innen und älteren Bürger*innen entwickelt und umgesetzt werden. Unter anderem wurden informelle und formelle soziale Netzwerke gestärkt und inklusive Strukturen entwickelt [11, 12].

## Methodik

Im Verbund PartKommPlus dienten halbjährliche Kolloquien, an denen in unterschiedlichen Zusammensetzungen Beteiligte aus den Teilprojekten teilnahmen, dem Austausch und der gemeinsamen Bearbeitung methodischer und inhaltlicher projektbezogener sowie projektübergreifender Fragestellungen. Aus der Selbstverständigung als gemeinsam Forschende in einem Forschungsansatz, dessen methodologische und epistemologische Grundlagen vergleichsweise wenig etabliert bzw. „kanonisiert“ sind, entstanden Fragen nach den Rollen der professionell Forschenden und der Co-Forschenden. Ebenso sind forschende Rollen für Praktiker*innen in Deutschland bisher wenig etabliert.

Entsprechende Fragen wurden einerseits in den Projektteams bearbeitet und andererseits bei den Verbundkolloquien teilprojektübergreifend in themenbezogenen Workshops – so auch zur Frage der Rollen – von Beteiligten anhand projektbezogener Erfahrungen und Analysen diskutiert; vorläufige Antworten wurden erarbeitet. Aus Transkripten entsprechender Teambesprechungen (AH4 1, 30.08.2016) und Workshops (WS1 20.02.2017, WS2 19.09.2017) wurden die Perspektiven der verschiedenen Beteiligten auf die Rollen und ihre Dynamiken mithilfe einer Globalauswertung [13] destilliert und inhaltsanalytisch ausgewertet. In diesem Rahmen wurden in einem gemischt deduktiv-induktiven Vorgehen Kategorien gebildet. Somit wurden theoretische Vorüberlegungen der Wissenschaftler*innen bei der Auswahl der Bedeutungsaspekte berücksichtigt (deduktiver Anteil); gleichzeitig konnten die subjektiven Sichtweisen der Beteiligten gegenstandsnah erfasst werden. Der hierauf basierende Diskussions- und Schreibprozess wurde zunächst weitgehend von den Wissenschaftler*innen bestritten und durch Feedbackschleifen mit den beteiligten Praxispartner*innen erweitert und validiert.

## Ergebnisse

Ausgewählte Befunde werden im Folgenden anhand von 5 zentralen Thesen entwickelt; diese wurden in Auswertungsworkshops von Wissenschaftler*innen und Praxispartner*innen (mit W und P gekennzeichnet) gemeinsam erarbeitet.

### These 1.

Rollen im Kontext von PGF entwickeln sich aus den jeweiligen Bedingungen im „Feld“ und den damit verbundenen Ressourcen, Kompetenzen sowie den persönlichen und beruflichen Interessen. Praxispartner*innen und Wissenschaftler*innen sind an ihre jeweiligen Felder und ihre dortigen Rollen (Routinen, Verpflichtungen, Interessen, Ressourcen) gebunden.

Wissenschaftler*innen und Praxispartner*innen sind an Vorgaben und Logiken ihrer jeweiligen primären professionellen Rollen gebunden. Inwiefern sie diese Rollen verlassen und gemeinsam forschen und handeln (können), hängt u. a. davon ab, wie stark sie mit ihren primären Rollen identifiziert sind und welche Handlungsspielräume sie haben bzw. wahrnehmen.

Unter günstigen Umständen werden bei potenziellen Praxispartner*innen Erwartungen und Interesse geweckt, für die eigene Tätigkeit und die eigenen Ziele profitieren zu können.

… logisch, machen wir sofort mit! Ja? Weil … weil ich einfach die Chancen gesehen habe. … ich habe nur gesehen, Mensch! Was kannst du denn daraus machen? … und das trägt bis heute ja durch (P Verwaltung, WS2 1008-18).

Positionen, Aufgabenzuschnitt und Organisationskultur können der Annahme von „fremden“ Aufgaben entgegenstehen. Für umfassendere Tätigkeiten außerhalb der zugeschriebenen Zuständigkeiten benötigen Praktiker*innen ggf. eine entsprechende Weisung bzw. Erlaubnis. Das Angebot, zu Co-Forschenden zu werden, kann auch Irritationen auslösen, wenn diese Rolle als unvereinbar wahrgenommen wird, weil sie bspw. im Konflikt mit primären Aufgabenbeschreibungen steht oder weil ein durchorganisiertes Arbeitsfeld nur geringe Handlungsspielräume zulässt. Insofern wird eine solche Möglichkeit nicht immer wahrgenommen.

Wir haben das ja auch ganz anders erlebt, dass es eben nicht so stattgefunden hat, … wie jetzt die [XY], die hat diese Rolle für sich nicht angenommen. Die … hat es abgelehnt. … ja, und auch die [YZ] haben eigentlich quasi mitgemacht, … aber die sind bei einer bestimmten Stufe dann auch … ausgestiegen. … Sie sind quasi ihrer Rolle treu geblieben und wir sind unserer Rolle treu geblieben. Also da gab es keine Bewegung, würde ich mal sagen (W, WS2 1683-98).

Bürgerschaftlich engagierte Praxispartner*innen sind u. U. Verbindungsglied zwischen Community, Praxispartner*innen und Wissenschaftler*innen. Sie können Weiterentwicklungsmöglichkeiten für sich sehen, die auch das eigene (zukünftige) Leben konkret betreffen können:

Ich möchte in diesem Ort … beruhigt alt werden und will vielleicht ein bisschen versorgt alt werden, wenn es sein muss. Aber ich möchte auch eine Situation vorfinden, wo ich sagen kann: Es ist vielfältig, also man kann nicht alles haben, aber wo ich eine Auswahl habe. … Und wenn es das nicht gibt, dann mache ich erst recht mit …, um daran zu drehen und zu arbeiten (P Zivilgesellschaft, AH4 1 124-131).

Wissenschaftler*innen in PGF wiederum begeben sich in die Praxisfelder, um dort an den gemeinsam entwickelten Zielen mitzuarbeiten; sie bleiben gleichzeitig dem Wissenschaftssystem verpflichtet. Sie organisieren Reflexionsräume (wie bspw. die hier ausgewerteten Workshops), um die gemeinsame Praxis zu analysieren, aber auch um Daten für die Publikationen zu gewinnen, die aus den gemeinsamen Erkenntnisprozessen entstehen und zumindest teilweise den Regeln des Wissenschaftsbetriebs folgen müssen.

Ich denke, da sind wir in der Verantwortung … ein Manuskript halbwegs einreichungsfähig zu machen. … Und ihr [P angesprochen] wenn ihr wollt, da dran noch mal weiter feilt, also sozusagen die Sicht der Praxispartner extra [darstellt]. Aber den Prozess … den haben wir im Auge und den sollten wir dann auch klar strukturieren und transparent machen (W, WS2 2013-2021).

Das, was Forscher so machen [lacht] (P Verwaltung, WS2 2043).

### These 2.

Durch wechselseitige und gemeinsame Entwicklungs- und Erkenntnisprozesse verändern sich die Rollen im Forschungsprozess.

Zu Beginn partizipativer Projekte müssen Wissenschaftler*innen – in entsprechend angepasster Sprache – viel erklären und werben, weil sie aufgrund des partizipativen Ansatzes wenig konkretisierte Zielsetzungen mitbringen, die zeitlichen Anforderungen hoch sind und für die Co-Forschenden der Forschungsaspekt zunächst oft ungewohnt ist.

… am Anfang des Verfahrens …, das habe ich bemerkt, dass die Wissenschaft mit ihren Forschungszielen der Verwaltung gegenüber sehr viel tun muss, um es zu erklären, um eine Mitarbeit herauszufordern …. Aber ich glaube, wenn man so ein Projekt neu startet, egal, wie, wo, dann muss die wissenschaftliche Seite … auch vielleicht in verschiedenen Redewendungen, Redearten das rüber bringen, denn in einer Kommune … ist der Querschnitt der Bevölkerung, wenn es gut aufgestellt ist, ne (P Zivilgesellschaft, WS2 959-73)?

Die Identifikation mit der partizipativen und damit gestaltenden Forschungsrolle entwickelt sich anhand von Erfahrungen und sich verändernden Vorstellungen über Forschung. Gleichzeitig wächst das Bewusstsein bei Praxispartner*innen, dass damit Zeit und Arbeit verbunden sind und Kapazitäten und Spielräume erforderlich werden.

Und wenn ich jetzt mit forsche, dann habe ich ja auch ein eigenes Erkenntnisinteresse; das funktioniert aber auch nur, wenn ich selbst was reinbringe. … Das geht auch nur, wenn ich Ressourcen zur Verfügung habe. Ja, das wäre für mich so ein wichtiges Kriterium noch mal bei der partizipativen Forschung, was man mit berücksichtigen müsste für weitere Ausschreibungen … (P Verwaltung, WS2 1128-32).

Mit den primären beruflichen Rollen der Wissenschaftler*innen und verschiedenen Praxispartner*innen sind spezifische Kompetenzen und Kenntnisse verbunden, die sie für die jeweils anderen nutzbar machen und ihnen damit Lernprozesse ermöglichen. Beide Seiten entwickeln Kompetenzen und übernehmen Aufgaben aus dem jeweils anderen Bereich. Im folgenden Zitat wird das Bewusstsein der Praxispartner*innen, dass beide Seiten lehrende Rollen übernehmen, sehr deutlich.

… wir sind irgendwie Experten für unseren Bereich, ihr seid die Experten in euren Bereichen [an W gerichtet]. Ihr erklärt … uns euren Bereich, ja und nur ich schreibe dazu nichts. [Lachen] Aber wir erklären es … ich sehe da ganz konkret ein Teilen … im Gespräch, an welchen Schwierigkeiten seid ihr gerade. Und ich versuche dann zu erklären und euch das zu erleichtern, woran liegt es gerade (P Verwaltung, WS2 1420-24)?

Rollen entwickeln sich in Interaktion; sie können sich – von den jeweiligen Personen und Konstellationen abhängig – mehr oder weniger konstruktiv und kooperativ ausbilden.

… schon dadurch, dass ihr [W] da drin seid, ändert ihr das Setting schon. Und … daher müssen sie [P] ihre Rolle auch neu definieren. Weil auf einmal ist jemand anderes … im Raum, der neben mir als Professioneller eben handelt. So, also und im besten Fall ist es ähnlich wie bei mir, ist es ein reflexiver Charakter, aber im schlechtesten Fall ist es eine Konkurrenzveranstaltung dann (P Verwaltung, WS2 1875-82).

Hier beschreibt ein Praxispartner der Verwaltungsebene seine Beobachtung, wie sich das Rollenverhältnis zwischen Wissenschaftler*innen und Praxispartner*innen auf Fachkräfteebene problematisch im Sinne einer Konkurrenzsituation entwickeln kann. Im positiven Fall entsteht hingegen eine reflexive Gemeinschaft, die Entwicklungs- und Veränderungsprozesse individuell und kollektiv ermöglicht und dabei Wissen über diese Prozesse generiert, also die Rollen von Forschen, Entwickeln und Umsetzen zeitweise miteinander teilt [14].

„Es ist eine Professionalisierung, eine Reflexion der eigenen Arbeit …. Weil es … für mich die Chance gab, wirklich genau hinzugucken.“ (P Verwaltung) „Genau, aber das ist zunehmend ein gemeinsamer Prozess geworden.“ (W) „Ja, das ist absolut ein gemeinsamer Prozess. Und diese Chance … hätten wir nicht gehabt im Arbeitsalltag … Und so kann man einfach mal sagen, so, wir machen hier einen Cut und wir sitzen vier, fünf Stunden zusammen und wir gucken uns das an. Wie funktioniert es? Warum funktioniert es … und das ist eine ganz hohe Qualität … Weil ja das … bei uns auch ganz viel bewirkt hat. Was wir auch nach außen transportieren“ (P Verwaltung; WS2 1993-2007).

### These 3.

Der Aufbau vertrauensvoller Beziehungen ist unabdingbare Voraussetzung für gemeinsames Reflektieren, Handeln und Forschen***.***

In partizipativen Forschungsgemeinschaften können Wissenschaftler*innen und Praxispartner*innen durch geteilte Erfahrungen über die Zeit füreinander zu Vertrauten und kollegialen Partner*innen werden, was einen gemeinsamen Reflexions- und Handlungsprozess ermöglicht bzw. intensiviert.

[Was wir] in beiden Forschungsprojekten so beobachtet haben, ist, dass eben Vertrauen in einen partizipativen Forschungsprozess eine ganz wesentliche Bedingung ist, damit die Zusammenarbeit klappt, es ist sozusagen ein Gelingensfaktor, der sich auch über den zeitlichen Verlauf der Forschung herausbildet und im besten Fall auch ein Stück weit intensiviert (W, WS2 165-8).

Und natürlich, je mehr Vertrautheit da ist, desto mehr hat man das Gefühl, ach, mit euch beiden kann ich darüber sprechen. Wir kennen uns lang genug. Weißt ja, wie er ist, ne? Ruf mal nächste Woche noch mal an oder so, weißt du (P Verwaltung, WS2 1429-33).

Ein so intensivierter Austausch ermöglicht vielfältige Erkenntnisse, die allen gemeinsam ein vertieftes Verständnis der jeweiligen lokalen Situation und der zugrunde liegenden Kontexte und dadurch das gemeinsame Handeln erlauben.

### These 4.

Die Vertrautheit der Beziehungen kann ein Dilemma im Hinblick auf die wissenschaftliche Ergebnisverwertung verursachen*.*

Vertrautheit lässt Vertraulichkeit entstehen. Einerseits laden vertrauliche Erzählungen oft zu einer vertiefenden Analyse geradezu ein; andererseits enthalten sie Informationen, die nicht veröffentlicht werden können, da eine Anonymisierung nahezu unmöglich ist.

Das bringt aber die Partizipative Forschung gleichzeitig in ein Dilemma. Denn im Rahmen dieses Vertrauensaufbaus besteht sozusagen eine vertrauliche Beziehung. Und im Rahmen dieser vertraulichen Beziehung werden relativ viele einschlägige Informationen zum Forschungsfeld ausgetauscht, und das … passiert meistens … in einem Kontext, der nicht klar als Forschungssituation deklariert ist. So. Und das führt natürlich zu diesem Dilemma, dass umso besser es eigentlich läuft mit diesem Vertrauensaufbau, umso mehr erfährt man, umso weniger kann man aber auch tatsächlich verwerten in diesem Forschungsprozess (W, WS2 168-76).

Rollen als Vertraute und kollegiale Partner*innen geraten also potenziell in Konflikt mit der Rolle der Wissenschaftler*innen, die analysieren und publizieren wollen und sollen. Für dieses Dilemma gibt es keine einfache Lösung – weil eine weitgehende Abstraktion von konkreten Vorgängen die Möglichkeiten dichter und differenzierter Beschreibung wesentlich reduziert.

### These 5.

Durch den Fokus auf Veränderung ist partizipative Forschung (im Feld) tendenziell politisch. Wissenschaftler*innen und Praxispartner*innen können damit zu Verbündeten werden.

Durch den Anspruch, Veränderungsprozesse insbesondere mit benachteiligten Gruppen zu initiieren, werden die Forschenden unabwendbar zu einem Teil des lokalen politischen Handlungsfeldes. Aus einem tradierten Verständnis von Forschung heraus wird der intervenierende Charakter von PGF leicht übersehen. Allerdings brauchen Wissenschaftler*innen für den Einstieg eine mehr oder weniger formalisierte Vereinbarung mit lokalen Schlüsselpersonen, um überhaupt Zugang zu relevanten Einrichtungen, Netzwerken oder Initiativen zu bekommen. Die Vereinbarungen enthalten Absprachen zu Zielsetzungen und sind in der Regel zunächst allgemein gehalten, um partizipative Entwicklungen zu ermöglichen. Sie sollten eine konstruktive Kooperation zumindest wahrscheinlich erscheinen lassen.

Wir suchen ja in dem Moment, wo wir Praxispartner suchen, auch Verbündete. … Ich kann mich noch an unser erstes Telefonat erinnern. Man tastet sich ja gegenseitig ab, ob das, was die Forscher im Kopf haben und was die Leute aus der Praxis im Kopf haben … ob das übereinstimmt. Also ob es sozusagen möglich ist, eine sinnvolle Koalition zu bilden. Und das bedeutet natürlich in der Tendenz … man verbündet sich mit bestimmten Leuten in diesem Feld von Praxis, was ja immer irgendwie ein lokalpolitisches ist, und natürlich stärkt man damit bestimmte Positionen und andere nicht (W, WS2 108-16).

Im Projekt Age4Health war dies bspw. die Einigung darauf, dass partizipative Prozesse mit Älteren niedrigschwellig aufgebaut und Fragen von Altersarmut mitberücksichtigt werden sollten. Im Projekt PEPBS ging es um Evaluationen innerhalb der Präventionskette in Braunschweig, die auf die Verminderung gesundheitlicher Ungleichheit von Kindern und Jugendlichen abzielt.

Deswegen muss … es frühzeitig auch thematisiert werden, welches Interesse denn die haben könnten, die also da zu Mitforschenden werden sollen. Denn ohne dass es … auch greifbar irgendwie ist, was der Nutzen ist … das macht niemand (P Verwaltung, WS2 1227-30).

Im Rahmen des Forschungsprozesses werden Beteiligte beabsichtigt oder unbeabsichtigt zu Bündnispartner*innen im Feld, wodurch bestimmte Akteure gestärkt, andere möglicherweise auch geschwächt werden. Lokale Diskurse können verändert werden bzw. an Momentum gewinnen. Konkurrenzsituationen können (ungewollt) erzeugt oder forciert werden. Inhalte und Ziele, die in den Projekten umgesetzt werden, können auch kommunalpolitisch durch die den Wissenschaftler*innen zugeschriebene Autorität ein höheres Gewicht bekommen; damit erfahren die entsprechenden Akteure und Positionen Unterstützung.

Das sieht die Forschung quasi von außen, dass das eigentlich irgendwie ein wichtiges Thema ist, das man bearbeiten müsste. … dass es schon so Themen gibt, wo ihr oder Sie selber sagen, das sind Missstände, oder Probleme, Sachen, die nicht gut laufen. Und die können wir jetzt selber vielleicht nicht publik machen, aber wenn das jemand anders auf eine bestimmte [Zustimmung] Art und Weise tut, könnte es mir auch nützlich sein vielleicht (P, WS2 1494-99).

## Diskussion

In diesem Beitrag wird eine Analyse der Rollen und Rollendynamiken zwischen Wissenschaftler*innen und Praxispartner*innen in der partizipativen Forschung präsentiert. Die Ergebnisse resultieren aus einer gemeinsamen Prozessanalyse der Beteiligten, die über mehrere Jahre gemeinsam als Steuerungsgruppen die Projekte Age4Health und PEPBS organisiert haben.

Die Ergebnisse zeigen, dass die Forschungspraxis der PGF die Rollen und die mit ihnen zusammenhängenden Beziehungen zwischen Wissenschaftler*innen und Praxispartner*innen sowie die daraus resultierenden Dynamiken anders als in traditionellen Forschungssettings prägt. Eine enge Zusammenarbeit geht mit dem Aufbau von Vertrauen einher. Im positiven Fall werden die Beteiligten zu Vertrauten und kollegialen Partner*innen. Eine forschende Haltung bildet die Grundlage vertiefter gemeinsamer Analysen des Handlungsfeldes und der initiierten Veränderungsprozesse und damit einer reflexiven Praxis [14]. Dies ermöglicht allen Beteiligten, zu Lernenden und Forschenden zu werden und damit die Grenzen ihrer gängigen Alltags- bzw. Berufsrollen zu erweitern. Hinsichtlich unterschiedlicher Kompetenzen, Ressourcen, Verantwortlichkeiten und Handlungsmöglichkeiten müssen die Rollenerwartungen und -gestaltungen benannt und ausgehandelt werden.

Erfolgreiche Kooperationen haben außerdem geteilte inhaltliche Grundhaltungen und Ziele als Voraussetzung. Intensives gemeinsames Forschen und Arbeiten (und damit die Verbindung der Rollen als Forschende, Quasi-Kolleg*innen und Bündnispartner*innen) ermöglichen tiefgreifende Erkenntnisse über lokale Zusammenhänge und Prozesse. Allerdings müssen Ressourcen auf beiden Seiten, d. h. Forschungsmittel und -unterstützung auf der wissenschaftlichen Seite sowie Zeit und Entscheidungsspielräume auf der Praxisseite, abgesichert sein. Das bedeutet auch, dass PGF nicht überall, wo man sie als sinnvoll ansehen könnte, initiiert werden kann. Zudem können die gewonnenen Erkenntnisse nicht immer vollständig für entsprechende Analysen und Publikationen nutzbar gemacht werden, da notwendige Anonymisierungen wissenschaftliche Verwertung zumindest in Teilen verunmöglichen.

Wir haben uns hier, begründet in den Konstellationen unserer Projekte und in den zum Zeitpunkt der Konzeptionierung des Artikels zur Verfügung stehenden Daten, auf Aspekte der Rollendynamiken zwischen Wissenschaftler*innen und Praxispartner*innen beschränkt. Wenn man auch Forschungsprozesse betrachtet, in denen Community-Partner*innen eine stärkere Rolle einnehmen, ist davon auszugehen, dass weitere Rollen und Dynamiken sichtbar werden.

Die Begrenzung auf 2 Projekte, beide im Kontext kommunaler Gesundheitsförderung, ist als Limitierung anzusehen, da die Ergebnisse damit nicht hinreichend empirisch gesättigt sind. Sie können jedoch als Hypothesen und damit als Ausgangspunkt für weitere Reflexionen und Analysen von Rollendynamiken in der PGF betrachtet werden. Mit einer erweiterten Datenbasis könnte die Beschreibung möglicher Rollen und ihrer Dynamiken weiterentwickelt werden. Auch könnten Zusammenhänge zwischen Beziehungen und Rollen theoretisch und empirisch weiter untersucht werden. Hier zeigen sich vielversprechende Ansatzpunkte für anschließende Vorhaben.

## Fazit

Die Perspektive auf die Entwicklung von Rollen in der PGF ist relevant, um fördernde und hemmende Bedingungen sowie Erfolge und Misserfolge von partizipativen Projekten zu verstehen. Erwartungen von Wissenschaftler*innen und Praxispartner*innen an sich selbst und die jeweils Beteiligten sollten deshalb von Anfang an und im weiteren Verlauf transparent ausgetauscht und fortlaufend reflektiert werden; eine klare Zuschreibung ist nur in Teilen sinnvoll und möglich. Die hier dargestellten Prozesse zeigen, dass die Reflexion der Rollengestaltung einen eigenen Raum benötigt und von operativen Prozessen zu trennen ist.

Durch den Fokus auf Veränderungen in den jeweiligen Handlungsfeldern werden potenziell Interessen der Akteure im Feld berührt; die aus diesem Fokus resultierende Parteilichkeit (bspw. für benachteiligte Gruppen) muss kommuniziert und in ihren Auswirkungen kontinuierlich verhandelt und reflektiert werden.

Die Entwicklung konstruktiver und kooperativer Rollen des Forschens, Lernens und Handelns beruht auf einem Grundstock geteilter Werte, Ziele und Interessen, die bestenfalls vor Beginn geklärt und vereinbart werden sollten.

## Footnotes

1 Wir gehen dabei von einem konstruktivistischen Rollenverständnis aus, in dem Rollen kreiert, gestaltet und verhandelt werden und nicht – wie in einer funktionalistischen Perspektive – mehr oder weniger vorgegeben sind und von Individuen „gespielt“ werden. Wir verstehen Rollen als ein Set von Aktivitäten und Haltungen, das zwischen Akteuren eine geteilte Realitätssicht erzeugt. Es handelt sich um Idealtypen, die aber sozial konstruiert und daher auch veränderlich sind und so zwischen Stabilität und Veränderung vermitteln [1]. „‚Gelebte‘“ Rollenverhältnisse sind durch institutionelle wie auch durch informelle persönliche Elemente charakterisiert.

2 PartKommPlus untersucht als partizipativer Forschungsverbund, wie integrierte kommunale Strategien der Gesundheitsförderung entwickelt und umgesetzt werden können (www.partkommplus.de).
